# A Study into the Effect of Different Nozzles Shapes and Fibre-Reinforcement in 3D Printed Mortar

**DOI:** 10.3390/ma12101708

**Published:** 2019-05-26

**Authors:** Pshtiwan Shakor, Shami Nejadi, Gavin Paul

**Affiliations:** 1 Centre for Built Infrastructure Research, School of Civil and Environmental Engineering, Faculty of Engineering and Information Technology, University of Technology Sydney, Ultimo, NSW 2007, Australia; 2 Centre for Autonomous Systems, School of Mechanical and Mechatronic Engineering, Faculty of Engineering and Information Technology, University of Technology Sydney, Ultimo, NSW 2007, Australia

**Keywords:** extrusion printing, cement mortar, mechanical properties, fibre reinforced printed object

## Abstract

Recently, 3D printing has become one of the most popular additive manufacturing technologies. This technology has been utilised to prototype trial and produced components for various applications, such as fashion, food, automotive, medical, and construction. In recent years, automation also has become increasingly prevalent in the construction field. Extrusion printing is the most successful method to print cementitious materials, but it still faces significant challenges, such as pumpability of materials, buildability, consistency in the materials, flowability, and workability. This paper investigates the properties of 3D printed fibre-reinforced cementitious mortar prisms and members in conjunction with automation to achieve the optimum mechanical strength of printed mortar and to obtain suitable flowability and consistent workability for the mixed cementitious mortar during the printing process. This study also considered the necessary trial tests, which are required to check the mechanical properties and behaviour of the proportions of the cementitious mix. Mechanical strength was measured and shown to increase when the samples were printed using fibre-reinforced mortar by means of a caulking gun, compared with the samples that were printed using the same mix delivered by a progressive cavity pump to a 6 degree-of-freedom robot. The flexural strength of the four-printed layer fibre-reinforced mortar was found to be 3.44 ± 0.11 MPa and 5.78 ± 0.02 MPa for the one-layer. Moreover, the mortar with different types of nozzles by means of caulking is printed and compared. Several experimental tests for the fresh state of the mortar were conducted and are discussed.

## 1. Introduction

Automation processes have contributed significantly to industrial fabrication and economic aspects of production. The construction industry has limited automation when compared with other industrialized sectors, such as manufacturing. By developing additive manufacturing processes, construction companies could potentially increase revenue and productivity, whilst simultaneously improving safety. There is evidence that automated machinery plays a crucial role in regulating time management and is less costly and more eco-friendly [1,2,3,4,5,6].

**Figure 1:**
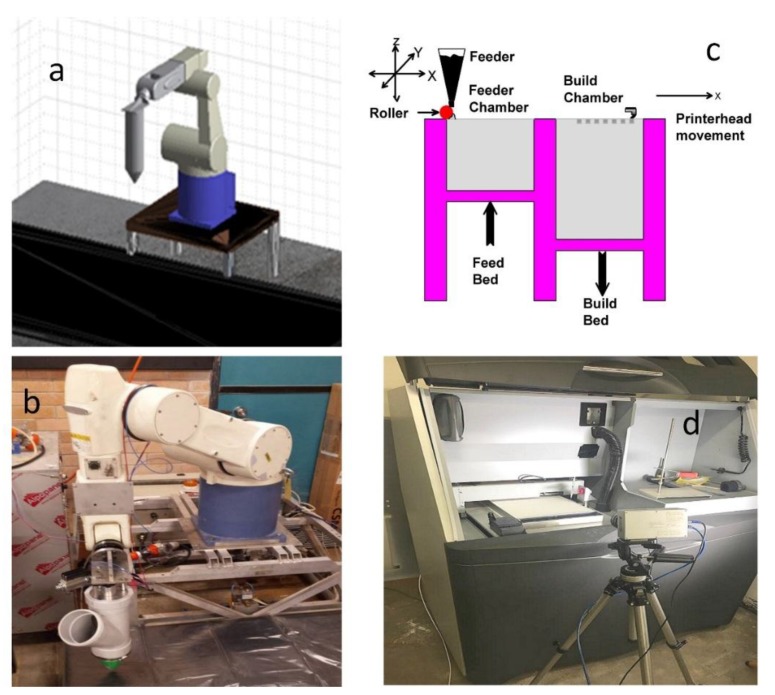
(**a**) Robot simulation, with the nozzle assembly attached, used to demonstrate the motion plans and check for potential collisions or undesirable paths; (**b**) a real-world robot with attached auger nozzle assembly; (**c**) schematic demonstration of the inkjet 3DP process; (**d**) real inkjet 3DP.

It is obvious that Additive Manufacturing (AM) can play a positive role in construction applications. In AM, the two most popular techniques of three-dimensional Printing (3DP) are ink-jet printing (powder-based or binder jet) and extrusion printing/plotting (extrusion-based process). They are the most widespread techniques in construction [7]. These two techniques are shown in Figure 1. Particularly, the AM for 3DP powder-bed process has been divided into three sectors have been recognized [8], (i) selective binder (cement) activation, (ii) binder jetting and (iii) selective paste intrusion.

The extrusion printing technique consists of an extruder which extrudes cementitious slurry through a nozzle attached to a frame to print a layered structure. The first example of this technique in the construction field was Concrete Printing which was proposed by Le et al. [9,10], and Contour Crafting established by Khoshnevis et al. [11].

The inkjet 3DP technique can be utilised for construction purposes. Christ et al. [12] incorporated polyacrylonitrile fibre fillers into the printed gypsum element to fabricate a reinforced scaffold. Shakor et al. [13,14] modified the original materials of the inkjet 3DP to cement mortar materials and found the *w/c* ratio of the printed materials.

Most recent studies on the fibre reinforced printed mortar and concrete for the 3DP technique, the lightweight cellular composite structures [15], the epoxy-based ink was used to enable the printing of cellular composites with a high-aspect ratio of fibre reinforcement to make a hierarchical structure using balsa wood. There are a few recent studies for extrusion-based 3DP on fibre-reinforced cementitious materials [16], which investigated mixing Portland cement with short fibres of carbon, glass, and basalt (3–6 mm). The mix design consisted of 61.5% weight of Portland cement, 21% of silica fume, 15% of water, and 2.5% of the water reducer agent. The retarder (Pantarhol 85, Pferrer, EN 934-2, Augsburg, Germany) was added (0.3% by weight of a hydration inhibitor) to reduce cement thickening while printing. The results of this study showed that the highest flexural strength was 30 MPa and compressive strength was 80 MPa. Another study that has been worked on the strain-hardening cement-based 3DP composites with high-density polyethene microfibers (HDPE) with a length of 6 mm and diameter 0.012 mm [17]. They found that 1% and 1.5% of HDPE fibre suitable for the printed application with notice of the 1.5% HDPE fibre in the cement-base created higher strain capacity higher and could distribute uniformly.

In the study by Buswell et al. [7], it has mentioned that the different shape of extrusion nozzles used in earlier works (i.e., circular, ovular or rectangular) but it did not provide many details for differences among the nozzles. Another study discusses that to attain the buildable layers and required shape, it is required to have an appropriate nozzle [18,19]. They mentioned that the nozzle orientation and various shape of the nozzle (i.e., circular, ellipse, square, and rectangular) has been used and argues that the circular nozzle provided more freedom and ease to change the nozzle angle for the printed part [18]. The study by Kwon [4] concluded that the square orifice or nozzle has a better surface finish than the ellipse type. Another study by Lim et al. [20] has been used the circular nozzle with various diameters of 4–22 mm, then the optimum diameter for their study was 9 mm.

Only a few studies covered concrete mix proportions for 3DP in the literature. However, one of the studies that used concrete mix printing through the extrusion methodology is the study of Le et al. [21]. They prepared a cement-based mortar consisting of silica fume, cement, sand, polypropylene fibre, and fly ash as the main ingredients. The optimum concrete mix design for the study was shown to be silica fume 83, cement 579, sand 1241, fly ash 165, and water 232 (kg/m^3^). Le et al. [21] scrutinized the mechanical properties of high-performance printed concrete by using a 9 mm diameter nozzle. The resulting compressive strength in different directions was between 75 and 102 MPa for the printed samples. Researchers have also used gypsum Lim et al. [20], where the mix of gypsum and cement are optimised for the 3DP, with experimental tests of compressive strength of 100–110 MPa being achieved. Lim, Buswell, Le, Wackrow, Austin, Gibb and Thorpe [20] used the short delivery method by placing the hopper on the top of the extruder. They also used the small batches of materials to control the hardening of the mix. There is a study on the 3DP earth-based materials using alginate as a fast setting binder by means of the 6 DOF robot to print circular shape and rectangular shape earth-based samples [22]. They observed voids in the circular cross-section; contrarily, the rectangular cross-section showed absence of cavity and voids. It also noticed that the compressive strength in the rectangular cross-section is higher than the circular cross-section.

The earlier study by Gosselin et al. [23] used two peristaltic pumps, one for the premixed slurry and the other for the accelerator agent. They have used different delivery methods and different mixing processes which have a significant effect on the printed product in terms of workability, shapability, and mechanical strength. Le, Austin, Lim, Buswell, Gibb and Thorpe [9] have used a hopper conveyed by a pipe to the CNC machine to print the slurry of the concrete. They faced issues regarding the cohesiveness of the mixing, such as high sand content.

In the present paper, we discussed an investigation of the delivery system to print the objects. As a result, the current study investigated the use of different delivery conveyors and their effect on the final result of the fabricated concrete.

The present paper also focused on validating the optimum mix proportion, which can be delivered using different delivery sources to print the cementitious slurry. The most suitable type of fibre, which flows easily with slurry, was selected. The study compared mechanical behaviours of the printed mortar with and without fibre-reinforcement. This study also discusses the benefit of fibre reinforcement into the mix design of the cementitious material. Fibre-reinforcement has been used in previous studies for different types of construction applications [17,24]. In this study, fibre filaments have been used to reinforce the cementitious mortar and improve the mechanical performance of the materials. In addition, different types of nozzles have been used and compared.

## 2. Experimental Program

### 2.1. Materials

Materials play a crucial role in 3DP of objects for construction applications. When the properties of the materials are optimised, the printing process will have a better result. In the present work, the materials consisted of fine sand (Sydney Sand, Sydney, NSW, Australia), ordinary Portland cement (General Purpose cement, Sydney, NSW, Australia) and chemical admixtures such as superplasticizer (SikaPlast^®^3 in 1, Sydney, NSW, Australia), accelerator (Sigunit L80AF, Sydney, NSW, Australia), and retarder (Retarder N, Sydney, NSW, Australia). The accelerator was used to accelerate the setting time of the mortar during printing while the retarder agent was used to open time and reduce the thickening of the cement in the delivery system and control rheology of the mortar [25]. Particle size distribution was conducted for the fine sand with a maximum particle size of 300 µm. The particle size distribution was prepared using a sieve mesh and particle size analyser. The fibre used was polypropylene (PP) (6 mm) (Elasto Plastic Concrete, Sydney, NSW, Australia), Table 1.

**Figure 2:**
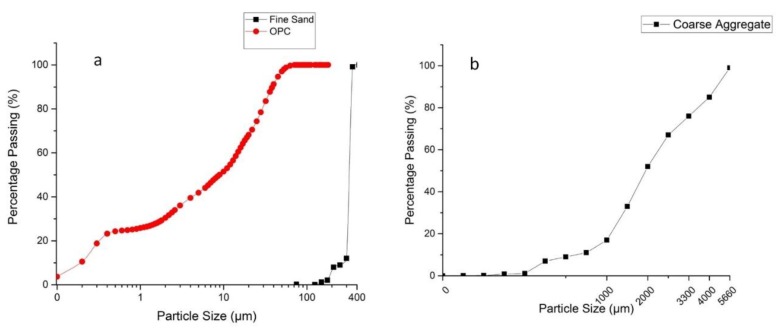
(**a**) Fine sand and Ordinary Portland Cement particle size distribution, (**b**) coarse aggregate particle size distribution.

Figure 2 shows particle size distribution versus the percentage of passing particles of ordinary Portland cement, fine sand, and coarse aggregate which were used in the mortar mix.

### 2.2. Design and Fabrication

#### 2.2.1. Extruder Adaptation and Delivery System

**Figure 3:**
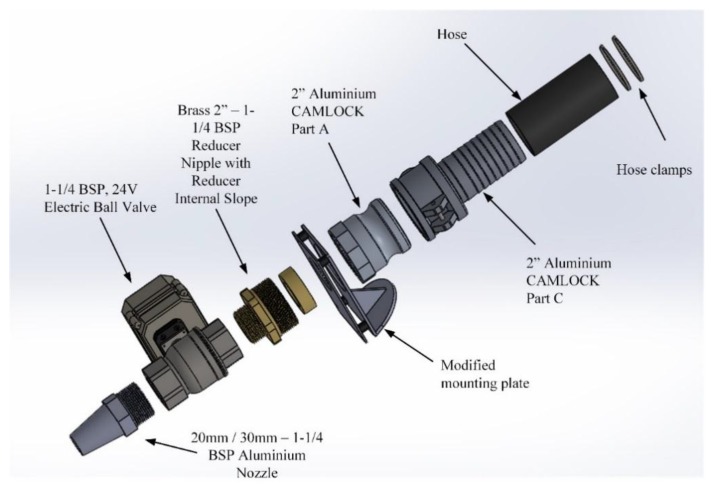
Exploded view of the extruder assembly that is connected to the progressive cavity pump and is attached to the end of the robot.

The extruder for the printed mortar shown in Figure 3 was designed so that it could be mounted to the end-effector of an industrial robot. The design, inspired by Anell [25], incorporates improvements to increase the efficiency of printing cementitious materials.

The pump was adopted to appropriately deliver material to the extruder. The extruder consists of the components shown in Figure 3. The end-effector tool is designed to easily adapt to the cavity pump. The following are design considerations while developing concepts for the new end-effector tool: (a) The tool must interface with the mounting bracket that is fixed to ‘Joint 6’ of the robot. (b) It must attach and provide a seal to the 50 mm hose connected to the cavity pump. (c) Create the components with standardised components and joints for versatility and maximise customisation potential. (d) Create a means to print 10 mm, 20 mm, and 30 mm widths for samples. (e) Implement a means to stop the flow of material during the robot’s transition between starting positions (i.e., a valve). (f) No more than a total weight of 10 kg can act on the end-effector at any time. (g) The nozzle material must be made of a strong material (e.g., aluminium) to resist pressure build up but still be lightweight.

In order to be a successful design, it would need to satisfy the above criteria. A major focus was to make the tool modular and adaptable so that new elements could be added later without having to redesign the whole tool. Most of the components were deliberately chosen to be of a standard size so they can be easily and inexpensively sourced from hardware shops. The following are the components, as graphically represented in Figure 3, that make up the end-effector tool:

A caulking gun was used with different sized nozzles (14 mm, 20 mm, 35 × 10 mm). The caulking gun has been used in previous work by Al-Qutaifi et al. and Soltan et al. [26,27]. The caulking was used as a mechanically actuated gun to print the cementitious mortar.

**Figure 4:**
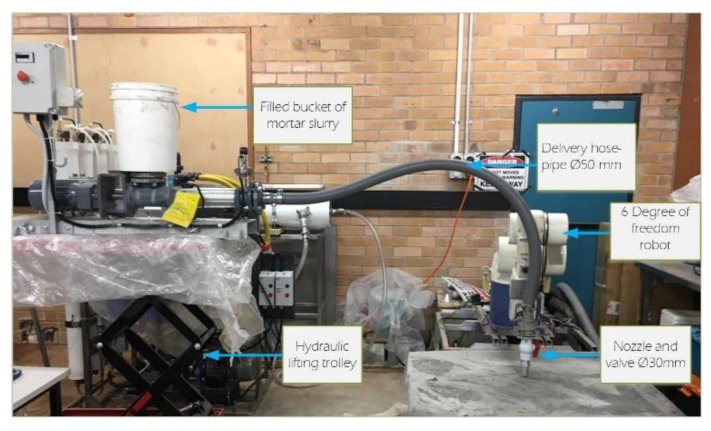
The final system with the progress cavity pump connected to the robot.

Figure 4 shows the connections of the pump to the end-effector. The progressive cavity pump connected to a tube with an inner hose with diameters of 20 mm and 30 mm, see Figure 4.

#### 2.2.2. Mix Designs for Cementitious Materials

Several trials for the cement mortar were prepared to check the optimum printable cementitious mortar. Fabrication and mix designs were examined based on previous investigations [28] and further mixed trials were implemented to improve smooth pumpbaility through the pump and delivery systems. Several studies were also prepared for concrete fabrication and printing members. Until now, 3DP concrete and mortar in the construction industry have not been technologically advanced enough and have not been investigated intensively on a large-scale.

The present study fills a gap in the literature by using different mixes until an optimum mix was obtained which could be more compatible with the different delivery system. This study also investigated a suitable delivery method for the printed mortar, while using different mortar mixes to obtain the optimum mix design. Two techniques were adopted to deliver the mortar, namely, using a caulking gun and a cavity pump.

The mix of mortar with various agents and chemical admixture has vital effects on the air entertainment in the mix, leading to a lot of residual air in the mix. Agents such as superplasticizer, accelerator, retarder and water reducer, affect the percentage rate according to the type of medium and the delivery system. However, for these mixes, the setting time and shapability of the mortar have been considered. The mixes have been selected based on the better shapability of print and buildability performance.

For the caulking gun (internal diameter of nozzle circular 14 mm, 20 mm, rectangular 35 × 10 mm), several trials are presented in Table 2. Mixing times of 5–6 min were found to be the most suitable. Due to the inconsistencies of extrusion, printing heights are taken as an average measurement for caulking gun printing. However, the printing height for mortar printing via a robotic arm has been fixed according to the diameter of the nozzle, where the diameter of the nozzle is equal to the height of the end-effector from the printed platform. Table 2 shows the selected mix proportion from previous work [28], which has excellent performance while printing.

## 3. Fresh and Hardened Properties Tests

### 3.1. Fresh Properties Tests

#### 3.1.1. Slump Test

The slump tests (spread-flow test) were prepared according to ASTMC1437 [29] to determine the flowability of the mortar mixes. Essentially, this test was conducted using a mini cone (100 × 70 × 50) mm. The first step of the slump procedure is pouring fresh mortar into the mini cone mould and then tamping it slightly, for settling down and levelling purposes, with a manual tamper for approximately 20 tamps.

The conical mould was then filled with the second layer and the third layer using fresh mortar. The second and third layers were poured and tamped afterwards using the same specified procedure as the first layer. Lastly, the additional paste on the top surface of the conical mould was scraped and flattened. After pouring three layers, the conical mould was lifted and then the height of the specimen was measured. Next, it was subjected to 25 tamps. The diameter of the spreading mortar was then measured in two perpendicular directions. Next, the value of relative slumps was determined using the following equation:

(1) $$rp={\left(d/d_{o}\right)}^{2}-1$$

where the *rp* is a relative slump, *d* is the average value of two measured perpendicular diameters of the spreading mortar, and *d_o_* equal 100 mm (which is the bottom diameter of the mini-cone).

#### 3.1.2. Squeeze Flow Test

**Figure 5:**
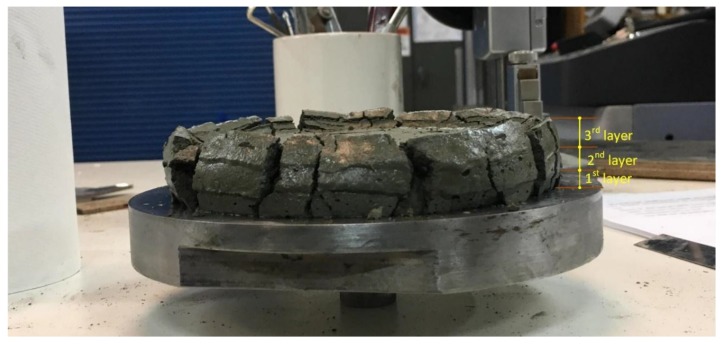
Triple layers of the mortar after squeezing under a uniaxial compression load.

The squeeze flow test was prepared according to the Brazilian test [30]. For this test, the uniaxial testing machine, Shimadzu (Shimadzu, Kyoto, Japan), was used to check the resistance of three main mixes (see Table 2) while in a fresh state. The displacement speed was 0.1 mm/second (see Figure 5). The surface roughness for this test was conducted using a portable profilometer (Taylor Hobson Surtronic 3^+^, Berwyn, PA, USA). This test was conducted for the single layers, double layers and triple layers.

#### 3.1.3. Setting Time

The Vicat test was used to validate the layer setting-time of the cementitious materials. In this study, a few trials of mortar mix (T5, T8, T12) were selected for a few of these trials and the outcomes are presented in the results section.

The measurement for the Vicat test was implemented according to ASTMC191-13 and Sleiman et al. [31,32]. The diameter of the needle was 1 mm and has a fixed weight of about 300 g on a movable rod. A sample of normal uniformity fresh mortar was prepared and placed in a container 40 mm in height. The test consisted of the measurement of the needle penetration to a required depth which falls due to gravity. The initial setting time was measured every 15 min. The first 15 min recording of the needle penetration was 39 mm ± 0.5 mm. The final time recorded less than 0.5 mm of penetration. The setting time test for each printing trial used for the caulking gun printing and the real height of the printed layer is listed in the results section. The outcomes and further interpretations are also presented in the results section.

### 3.2. Hardened Properties Test

Several experiments were conducted to assess the mechanical and hardened properties of materials after completing the print and once components have reached the hardened state. It is essential to print the small structural members before printing at a larger scale. These tests were prepared based on the mortar materials’ behaviours. The mechanical properties of the printed objects are derived by the measurement of the materials’ behaviours, which can be used to indicate the yield strength, deformation of the materials and resistance of the materials. Three samples were used for each of the trial and mix designs. Table 3 presents the number of samples and batches. The small hollow column with 7 layers was prepared, 3 samples with 1% PP fibre (caulking gun only Ø14 mm) and the other 3 without fibre. Then, after 28 days they were tested for compressive strength under the uniaxial testing machine (Shimadzu, Kyoto, Japan). The printed column was performed via a robotic arm (Denso) using a cavity pump as a delivery system. The printed hollow column was prepared without any time lapse between printed layers and without wet curing for the printed specimens.

The printed specimens of flexural strength and compressive strength were printed by delivering systems of the caulking gun and cavity pump.

#### 3.2.1. Compressive Strength Test

To evaluate the mechanical properties of various mortar mixes, a uniaxial compressive strength test was conducted to check the suitability of the sample under compression before printing the complete components. The cubic specimen dimensions (50 × 50 × 50) mm were cast using manual mixing according to the Australian standard [33]. The loading rate was 0.833 kN/s. The testing machine used was from Shimadzu (Shimadzu, Kyoto, Japan). The printed hollow column was also prepared with a delivery system of a cavity pump to print the whole structure.

#### 3.2.2. Flexural Strength Test

To assess the flexural strength of the specimens, a three-point bending test was applied to all the main mortar mixes [34]. The prisms, with dimensions of (160 × 40 × 40) mm, were cast for that purpose using manual mixing. The various layers were printed using a caulking gun for the three-point bending test. The caulking gun can print with the dimensions of: (160 × 24 × 18) mm for one layer; (160 × 24 × 36) mm for two layers; (160 × 24 × 54) mm for three layers; and (160 × 24 × 72) mm for four layers. All used a 20 mm circular nozzle. Thus, only the width and thickness of the sample changed. A similar prism was cast for one, two, three, and four layers for a circular nozzle of 14 mm and a rectangular nozzle (35 × 10) mm. Three samples for each printed layer were prepared. These batches were prepared both with fibres and without fibres and used different cured conditions see Table 3. The caulking gun has been used in the previous study by [26,27]. The uniaxial testing machine from Shimadzu (AGS-X 50 kN, Japan) was used for this test. The loading rate was 0.426 kN/min.

## 4. Results and Discussion

### 4.1. Mechanical Tests and Materials Properties

Mechanics of materials is dealing with the behave of materials which subject to stresses, the mechanics of materials can be investigated in the fresh state or hardened state. Further, the mechanical strength test in the presented paper was conducted to measure the fresh state slurry of the mortar and hardened state of the mortar.

There are two major factors that have a significant effect on the final shape result of the product are (a) the number of printed layers, and (b) the discharging of mortar slurry through different nozzle shapes and sizes.

**Figure 6:**
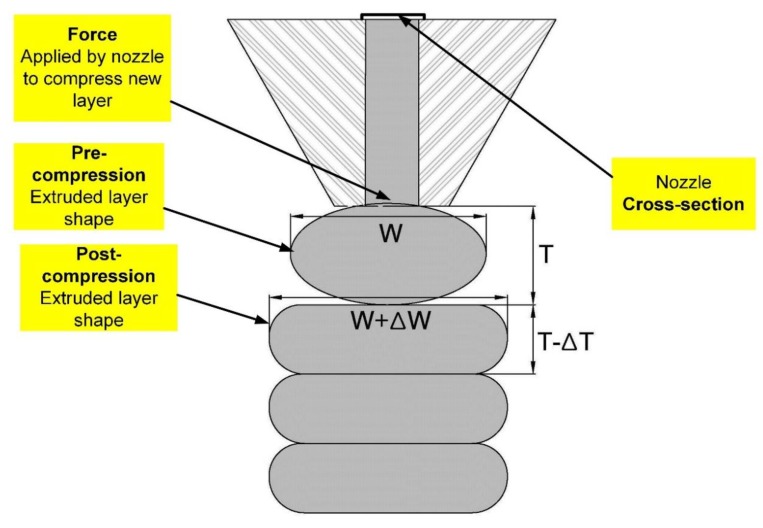
Schematic illustration of the prediction of the printed mortar passed through the circular nozzle.

**Figure 7:**
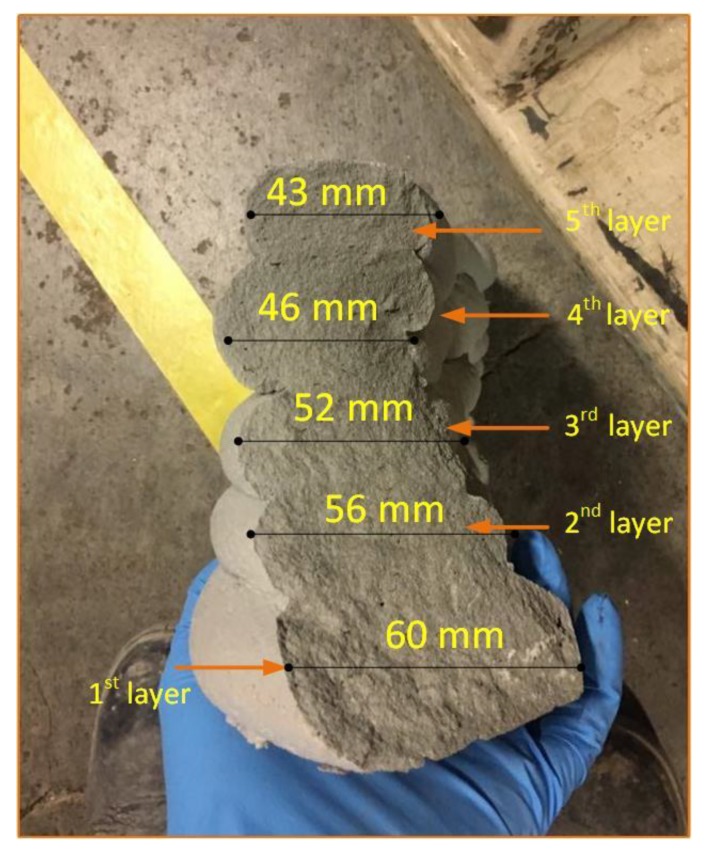
Cross-section of the details of the printed layers after crushing under a uniaxial compression load, using a cavity pump as a delivery system (nozzle Ø50 mm).

Figure 6 explains the change in width, which is specified as (*W +* ∆*W*), while the thickness is expressed as (*T* − ∆*T*). Figure 7 shows that the extruded layers have changed into their original shapes. The first printed layer would also change in width and thickness after loading the next layers. Once again, the first layer will face a change in its shape after the printing of the third layer. This change is possibly continuous until the shape has reached stability in its form and has set enough. The last layer of the component did not encounter any modifications and changes due to no further layers being added, so it retained its own shape, Figure 7. This is the nozzle, Ø50 mm, that has been used for the printed specimens in Figure 7.

Where *W* is the true width and ∆*W* is the trace width error. *T* is the true thickness and ∆*T* is the trace thickness error. Therefore, the area of the object will change at the pre-compression and post-compression stages according to the rheology of the mix proportions and the forced impact by the next layers which are printed. Figure 6 shows that the area of the layers will vary according to the printing height, nozzle types, the mixing time, and the setting time of the materials. Therefore, the true area of each cross-section printed layer is equal to (*W +* ∆*W*) × (*T* − ∆*T*).

However, there could be a different result when the time intervals between layers changes. When a slight decrease in the time intervals happens between layers the rate of penetration between layers increases due to the viscosity and thixotropy (shear thickening) properties in the concrete. This is consistent with research by [35], which found that the shear thinning of the concrete could be changed to shear thickening by adding superplasticizer to the paste of the cementitious materials. Shear thickening is defined as the proportion of the shear stresses to the viscosity of materials which can be increased gradually. This phenomenon emerged during the pumping process of the mortar [36]. The mortar had resisted downward pressure and the viscosity also greatly increased. For this to occur, a mixer in the hopper needs to make a consistent movement in the container. In this study, a different ratio of superplasticizer was used. This had a significant influence on the setting time of the mortar and the viscosity of the mortar. The ratio of superplasticizer to cement materials was (0.67% to 1%).

**Figure 8:**
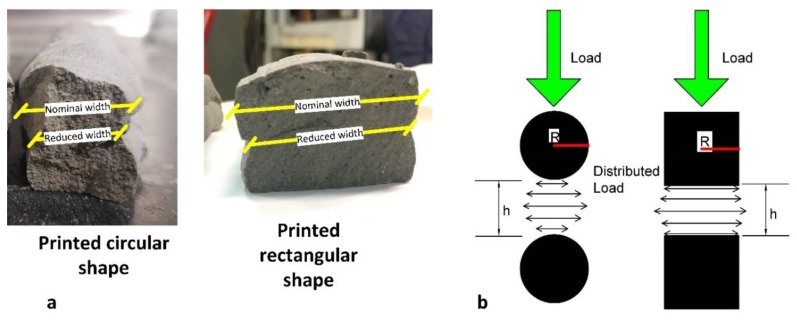
(**a**) Real printed circular and rectangular shape; (**b**) lubrication squeeze flow between two spherical particles (**left**) and two cubic particles (**right**) with an identical characteristic half-width, R (reproduced by Reference [38]).

The shape of the nozzles influences the printability, shape, and flowability of the slurry. The study, Li et al. [37] asserted that intercepting shocks are significantly changed according to the shape of the nozzles. The study found that square shaped orifices are faced with a higher interception than the other jets due to the four corners exiting at the nozzle. In addition, the penetration between two layers increases while the *w/c* and the number of layers increment proportionally. Moreover, the shape of the nozzles also affects the percentage of penetration between the two layers. If the nozzle shape is circular, the penetration rate increased slightly. This is due to less flatness of the previous layer which is laid down as a concave shape. The application of the spherical particles and square particles theory could be applied to the circle and square nozzles in terms of the shape and load applications. Figure 8 shows that the load in the square nozzles is distributed equally due to the radius of distribution in the square shapes. This distribution area is smaller in circular nozzles [38]. Böhmer et al. [39] found that in the inkjet printing technique the diameter of the droplet, which contains 0.3% polyvinyl alcohol solutions, would be larger than the diameter of the nozzle. They used a different concentration of polymer solution with the three different nozzle diameters. As a result, at a constant polymer concentration, smaller initial droplets were produced by the smallest nozzle diameter that, in turn, leads to smaller particles as well. Consequently, this could be similar to concrete slurry, where a higher flow of slurry is produced with increasing nozzle sizes.

Cwalina, Harrison and Wagner [38] stated that the particles with spherical and cubic shapes produce different results. The squeeze flow and load distribution between two cubic particles and two spherical particles with equivalent radii-lengths are illustrated in Figure 8.

For particles with an identical characteristic half-width, *R*, moving along their lines of the centre at a relative velocity, *V*, in a Newtonian fluid of viscosity, *ηf*, the lubrication force between the spherical and cubic particles is given, respectively, as [38]:

(2) $$F_{spheres}=\frac{6\pi V\eta fR2}{h}$$

(3) $$F_{cubes}=\frac{3\pi V\eta fR4}{h3}$$

In the two Equations (2) and (3), it is obvious that the reaction force between two particles increases when the shapes change from cubes to spheres. Therefore, this result will be similar for printing when square or circular nozzles are used. The printed shape will replicate the shape of the nozzles. The particles used in this experimental test were mostly spherical with some of the irregular shapes. As a result, the printed slurry will be a similar shape as it passes through the nozzle. The different shapes and sizes of the nozzles were also investigated in this study. The shapes used were circular and rectangular, with sizes of (20) mm in diameter and (35 × 10) mm. The forces were distributed evenly over the greater surface area in the square and rectangular shapes than in circular shapes. Consequently, the printed layers of the square or rectangular nozzle shapes withstand more layers than the circular nozzle shapes. It should be noted that the same mix ratio was used for the printed object utilizing different nozzle types. It was found that the nominal width in a rectangular shape was larger by 2 ± 0.85 mm than its reduced width (layer surface contact). Conversely, the nominal width of a circular shape was larger by 3.1 ± 0.75 mm than its reduced width.

Considering the forces applying to differently shaped particles, the higher forces emerged between flat cubic particle surfaces compared with the curved surfaces characteristic of spherical particles.

**Figure 9:**
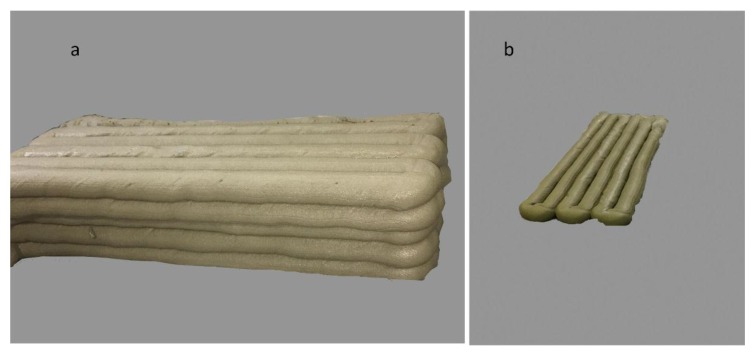
(**a**) Six layers of the printed object; (**b**) One layer of the printed object.

Figure 9 shows an object where one layer has six layers printed onto it. It has been printed to measure its dimensional geometry and test its mechanical behaviour.

**Figure 10:**
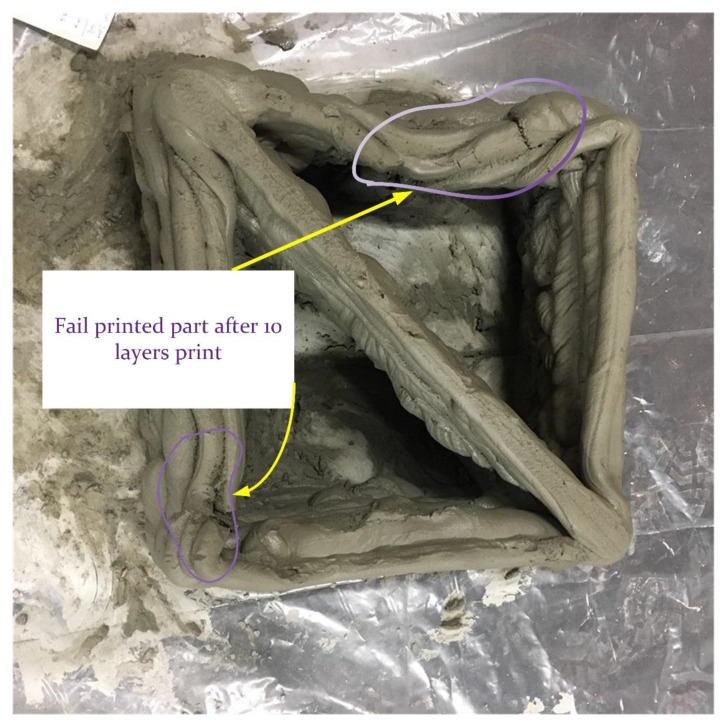
The movement of the robot arm has a major effect on the printing process, particularly when the height of the object increased to more than 7 layers.

For a printed object of over 120 mm (more than 7 layers), the oscillation at the arm of the robot increased in the end-tip of the arm which is most related to joint 4 and joint 6 in the robot [28]. Figure 10 shows how the printed layers collapsed after 10 layers of printing.

**Figure 11:**
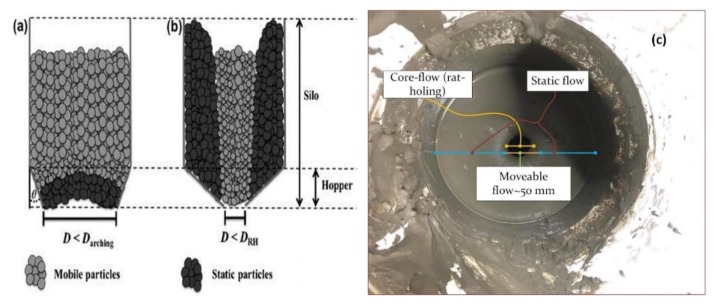
Schematic illustration of flow problems encountered in hoppers, namely, arching and rat-holing, during (**a**) mass-flow and (**b**) core-flow, respectively, where D = outlet diameter, D_arching_ = minimum arching diameter, DRH = minimum rat-hole diameter, and Ø = hopper half angle [40]. (**c**) Pictorial illustration shows the rat-holing flow in the bucket above the pump during pumping.

Another challenge that it faced during the printing process was the use of a flat-based hopper, as shown in Figure 11, where core-flow (rat-holing) occurred during the printing of the specimens. It is observed that some of the slurry close to the wall of the bucket is in a static state, while other parts of the slurry are in a mobile state.

**Figure 12:**
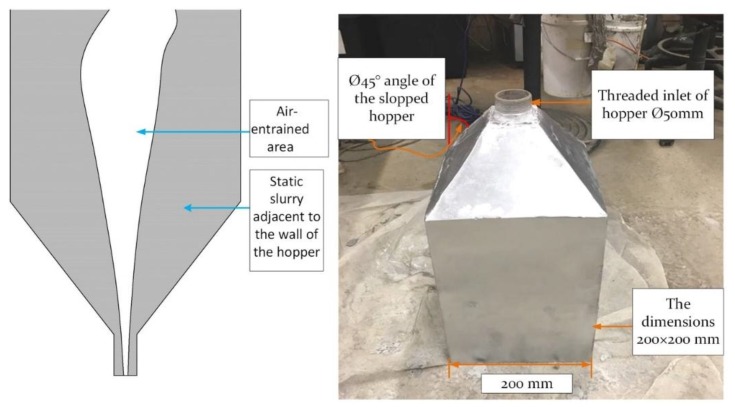
The hopper with an angle of Ø45° and the core-flow concept.

Figure 12 shows a modified hopper angle of Ø45° to improve the flowability in the hopper. Generally, fresh concrete or mortar during poured-in-place behaves as a liquid slurry (a viscoplastic fluid with high yield stress). However, the internal structure of slow casting concrete or when in a rest state leads it to flocculate. It also has the ability to resist the load from concrete cast over it without increasing lateral stress, despite the nature of the mould. Feys et al. [41] explained that the (hydro-) clusters are assembled together and become moulded from certain shear stress on the critical shear stress. By increasing the shear rate, the viscosity of concrete increases proportionally. This state of fresh concrete is called shear thickening. For the concrete properties, it is noticeable that when the temperature rises, the workability and slump of the concrete decreases. This is another reason that the mortar could not pass through the hopper effortlessly. Apparently, the longer concrete or mortar remains in the hopper, the more advanced the reaction and the higher the increase in temperature, which subsequently leads to an increase in the viscosity of the mortar. A temperature increase from 21–35 °C was measured after 30 min of the mixing process occurring.

Earlier studies explained the two types of powder flow pattern in the hopper: Mass-flow and core-flow [40]. The most noteworthy is the core-flow that emerges while feeding the concrete through the pump (deliver) to the robot. According to Fitzpatrick et al. [42], the moisture content has a high impact on powder particles in terms of flowability. Consequently, the surface forces between the powder particles or slurry and the wall surface play a major role in shaping the nature of the powder flow (see Figure 11).

Figure 12 shows a hopper with an angle of Ø45°, which reduced rat-holing but did not eliminate it completely. This rat-holing phenomenon happens due to the flocculates of the particles and maintains particles in the static state. Improving this situation requires consistent mixing in the hopper.

For most of the trials that were prepared in the experimental program, a set of prisms and cubes were prepared to validate the mechanical behaviour of the mixing property. The printed prisms have been created by selected trial 5 cementitious mortar as a mix of proportions. So, the printed mortar has been tested for each of the layers from (1–4) by caulking gun.

The outcomes suggest that waiting time between layers of 10 min is necessary, as it has been proofed by [43] and the ratios of water to admixture need further investigation.

**Figure 13:**
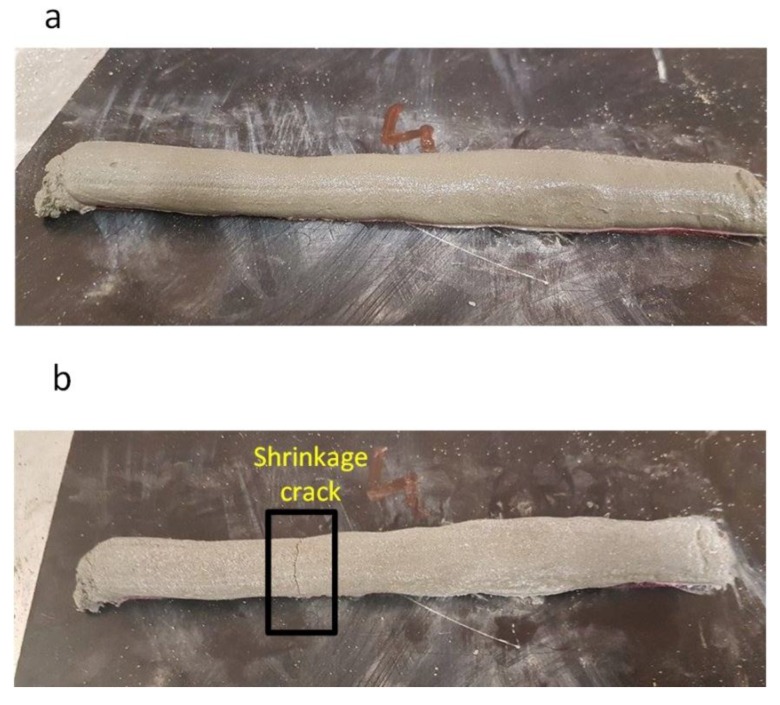
(**a**) The printed part immediately after printing; (**b**) the printed part after one-day drying.

The printed part is shown in Figure 13. Shrinkage cracks appeared on the printed part after one day of curing in the laboratory temperature. To reduce the cracks so the printed materials are stronger and exhibit fewer shrinkage cracks, chopped strand fibre was introduced.

#### 4.1.1. Slump Test

**Figure 14:**
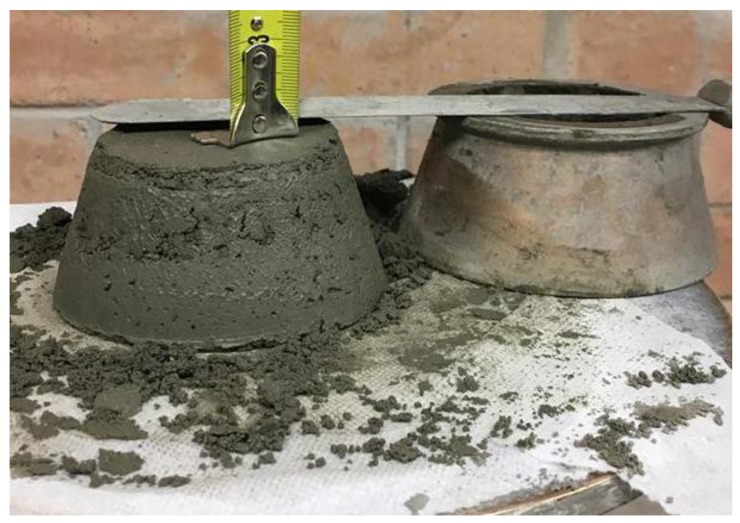
Mini-cone slump test for trial 8.

**Figure 15:**
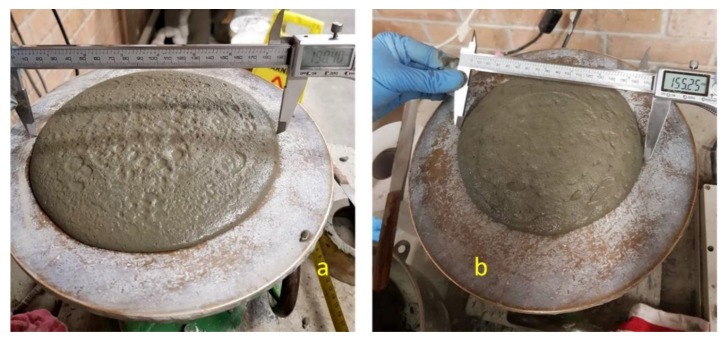
Spread-flow test: (**a**) For trial 5 which did not use fibre; (**b**) for the same trial by adding fibre with 1% polypropylene fibre.

This test showed the different results of the three different trial mixes which have three various slump results. This difference in results was based on their *w/c* mix ratio and the cohesiveness of their particles. Consequently, all the trial results have a different slump ratio. The deformation of each slump was 8.5 mm, 8.8 mm, and 12 mm for trials 5, 8 and 12, respectively. It is noteworthy that trial 8 has a greater slump than trial 5, with the difference likely due to the amount of *w/c* and superplasticizer in this mortar mix. It is also interesting that the mixing time and the time taken to pour into the slump have a great influence on the resulting mix. Each trial was, therefore, mixed for 5 min. As a result, trial 5 was expected to achieve higher penetration than trial 8, but trial 5 was more coherent than trial 8, as explained in the squeeze flow test, see Figure 14. Figure 15 shows the spread-flow test for trial 5 both in the presence and absence of 1% PP fibre.

Table 4 shows the results of the relative slump for all main mixes which were used for the printed samples. The minimum flow and slump occur in trial number 5 with 1% polypropylene fibre, which was expected due to the consistency and cohesiveness of this mix and the fibre content. The use of polypropylene fibre increased the mechanical strength of the structural element (Section 4.1) and it reduced flowability (Figure 15) and shrinkage (Figure 13). In addition, fibre increases the buildability of the mortar and stiffens the mass of the printed layer. For that reason, it is recommended that fibres be used in the printed specimens to increase stiffness and mitigate shrinkage in the printed part. Each test has been repeated three times.

#### 4.1.2. Squeeze Flow Test

**Figure 16:**
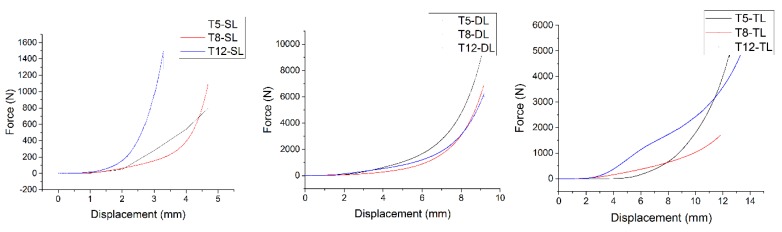
Maximum result of single layer (SL), double layer (DL), and triple layers (TL) for the trials (5,8,12).

The results of the squeeze flow tests showed different values in the single, double and triple layers for each of the selected trial mixtures (5,8,12). Each trial has been conducted 3 times. The maximum result of the three trials is shown in Figure 16. In the single layer mortar mix test, higher results were obtained in the reaction force value until it reached the required displacement. For instance, at a displacement of 2.99 mm, the required loads in trial 12 (concrete mix with small aggregate) were approximately 956.51 N. While at the same displacement for trials 5 and 8 (cement mortar), the load was approximately 277.82 N and 153.11 N, respectively. An examination of the results of the tests that utilised a double layer found significant differences in comparison to the single layer results. Trial 5 (cement mortar) had a reaction force which reached approximately 622.54 N when the displacement was about 3.99 mm, while for trials 12 and 8 the loads were 536.05 N and 275.75 N, respectively. At the same displacement, the results for the triple layers exhibited a similar pattern.

These results show that the mortar is more coherent than the concrete mixtures in the fresh state. Thus, it does not allow trapped air bubbles to remain in the mortar mixture. This is consistent with the study by Surendra et al. [44], who discovered that the mortar in the first 24 h had a greater compressive strength by comparison with normal concrete. Hence, a much higher percentage of open pores will exist in the concrete mixtures by comparison with the cementitious mortar due to the presence of aggregates. The larger particle sizes lead to a higher porosity within the concrete mixtures.

The reaction force is dependent on the number of chains (layers) and the force between the particles. Trial 12, a single layer test, showed that the presence of the small aggregate in the mixture can resist more force over the given displacement, Figure 16. Furthermore, Figure 16 shows that the mortar mix for more than one layer has better resistance than the concrete mix and that less penetration will occur between layers when the loads are applied Figure 16. It can be seen that for both double and triple layers, mortar has a better squeeze-flow performance.

#### 4.1.3. Setting Time

In Table 5, the setting time results for the different trial mixes is shown. There are minimal differences in the initial setting times of these trials.

The setting time has a crucial effect on the bond between printed layers and penetration rate between each printed layer.

The buildability tests, which depend on the setting time, have been applied for each trial by printing using an extrusion caulking gun.

Table 6 relates to trial 5; this trial was based on the original mix design, which used a 1:1 cement to fine sand ratio with a water/cement ratio of 0.33. This is able to be printed with the caulking gun and a circular nozzle of 14 mm diameter. The settling observed was, to some extent, expected with the relatively similar levels of sand and cement in the mix. Not much change was observed in the heights of the sample. Sample number 6 showed a failed extrusion as it had collapsed considerably.

#### 4.1.4. Compressive Strength Test

**Figure 17:**
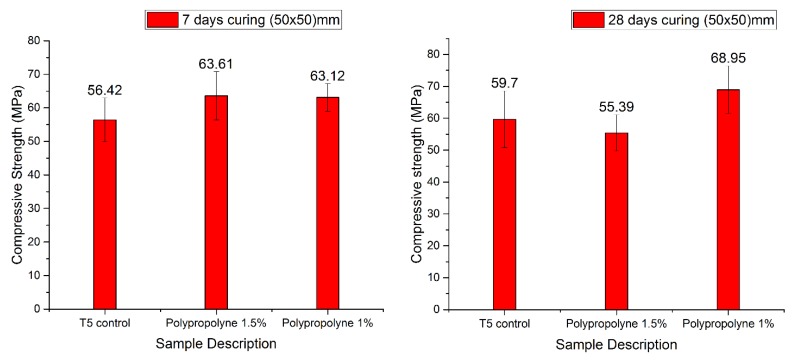
Compressive strength test results for manual mortar mix after 7-day and 28-day curing (the values denote the actual strengths).

For each of the optimum trials that were printed successfully, a uniaxial compressive strength test was conducted. Figure 17 presents the results of the test conducted for the manual concrete mix, which included polypropylene fibres with a different ratio and with a control sample.

**Figure 18:**
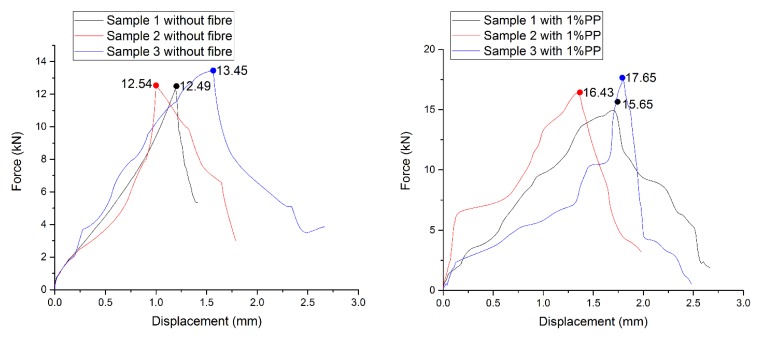
Results after 28 days for the short column (7 layers) without any time lapse between printed layers and without post-curing.

The compressive strength result for the 7 layers of the printed mortar is shown in Figure 18. It can be observed that the highest compression result for printed mortar without curing and after 28 days was 13.45 kN as a maximum load. The printed specimens were left in the control temperature room at 20 ± 2 °C without any additional post-curing. However, the compressive strength increased after using 1% of Polypropylene fibre in the mortar. The maximum strength was 17.65 kN after using fibre. Therefore, the increase in the percentage rate was 31%.

Figure 18 shows the trend of the printed hollow column (7 layers), it is clear from how the trend line (i.e., force versus displacement) varies and has an unstable trend line in both plots. This is more obvious in the printed specimens while using 1% PP fibre. The PP fibre significantly stiffens the structure during the early applied load on the hollow column. Therefore, a bump at the early stage of the printed sample can be observed.

**Figure 19:**
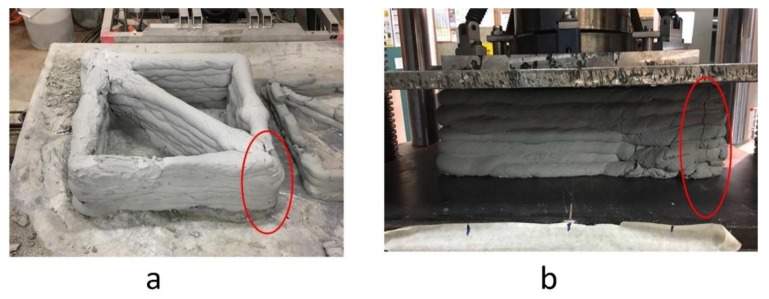
(**a**) The printed specimen after drying at lab temperature; (**b**) the printed specimen under uniaxial load.

Figure 19 shows the printed sample after drying at the control temperature in the lab. No shrinkage cracks or hairline cracks on the printed specimens resulted. After loading, cracks appeared on the edge of the printed column, as shown in Figure 19b. The results for the printed column showed that the average rate of the printed column is (12.83 ± 0.54 kN). This is equal to the strength of 2.37 MPa, however, this needs to be improved by post-curing and the use of large particle sizes. It is also obvious that the low strength was due to the number of layers. The printed column was a mortar mix rather than a concrete mix which normally has less resistance than normal concrete after curing for 28 days.

The short column under the uniaxial load cracked and ruptured at the edge of the sample, which showed the weakest part of the column. The edge of the column was revealed as the weakest part of the column due to the irregular movement of the robot while printing the column.

#### 4.1.5. Flexural Strength Test

**Figure 20:**
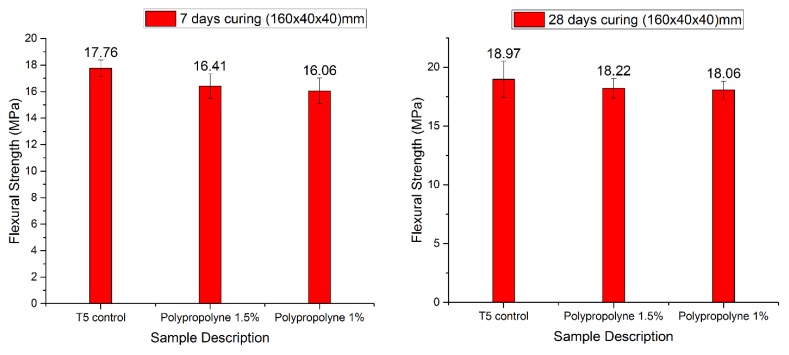
Flexural strength of manual mortar mix after 7-day and 28-day curing (the values specify the actual strengths).

The three-point bending test was applied for the optimum mix of mortar using a different type of fibre and different ratios. The flexural strength results for the different ratios are shown in Figure 20.

**Figure 21:**
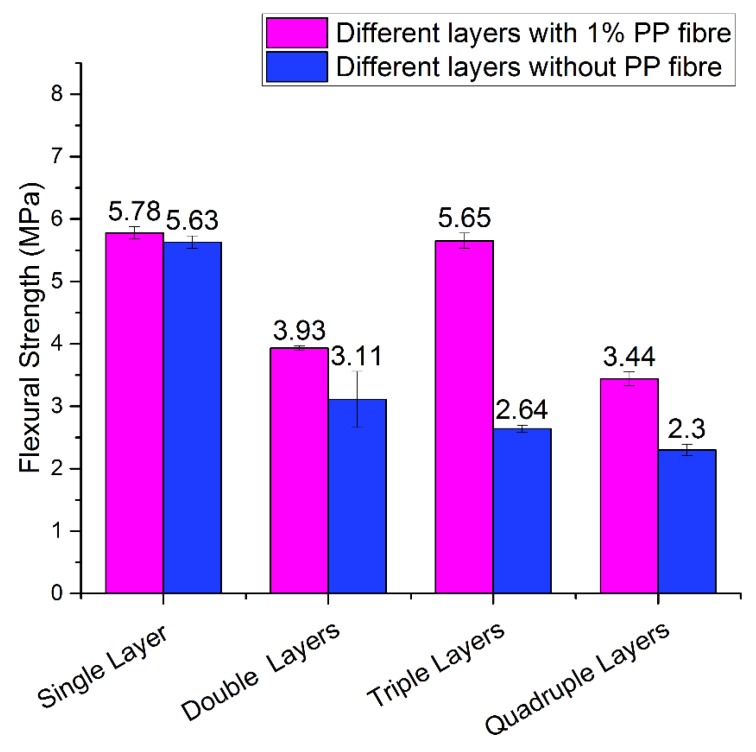
Flexural strength for the (1,2,3,4) layers of mortar mix with 1% PP fibre and without fibre for a circular nozzle of 14 mm after 28 days stored in controlled laboratories at the desired temperature.

The three-point bending test and flexural strength for the one, two, three and four layers of cementitious mortar with 1%PP fibre and without fibre are shown in Figure 21. The maximum result of 5.78 MPa for 28 days curing was observed in the single layer with 1% PP fibre content in the sample. Another high result was achieved for three printed layers which recorded 5.65 MPa. It is worth noting that using PP fibre increased the flexural strength in all variable layers. In addition, it is noticeable that when increasing the number of layers, the flexural strength decreases due to inconsistency, a high ratio of moisture content, and air trapped between layers. The flexural strength of the triple layers with 1% PP fibre is higher than the double layer printed specimens, this may be due to the inconsistency of the printed layers which resulted in higher flexural strength than a double layer. The reason for the flexural strength results being different among layers still requires further investigation. The moisture content on the printed surface layer, surface roughness, and orientational angle of bedded layers have a major contribution in the fluctuation of the results. Overall, the results show a decline in flexural strength as the number of layers in the printed specimen increases.

**Figure 22:**
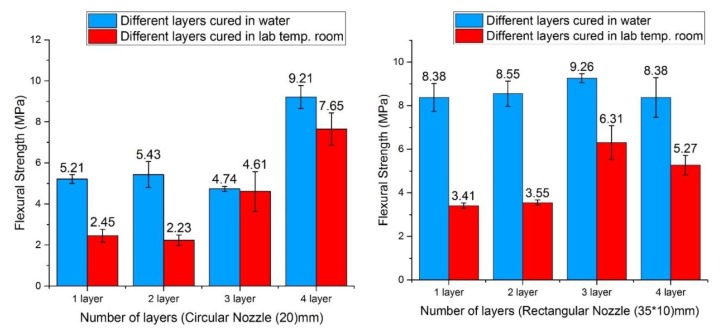
Flexural strength for the (1,2,3,4) layers of mortar mix for a circular nozzle (20) mm and a rectangular nozzle (35 × 10) mm at air temperature and with water curing.

Figure 22 shows the effect of different nozzles on the result of flexural strength. It is noted that the highest flexural strength is found in the 3rd printed layer of the rectangular nozzle when investigating the highest flexural strength in circular and rectangular nozzles in the wet medium cure. In Figure 22, all the layers of the rectangular nozzle have a similar result, but for the circular nozzle, each layer has unstable results. Therefore, it shows that the load distribution of the circular nozzle is reduced with the reduced surface area and width. This directly affected the mechanical strength results of the printed object (see Figure 8). Consequently, the result suggests that a rectangular or square shape has a constant result and a better result than a circular nozzle print. This is consistent with the study of Reference [22]. Conversely, it has been shown that wet medium curing is better than curing at air-temperature in vitro, as demonstrated by Reference [45], Figure 22. In addition, it is highly recommended to use fly ash in the mix to reduce the voids between particles and increase the durability of the mortar [46].

The caulking gun and cavity pump show differences in the printed specimen results due to differences in the way fresh state material is deposited, e.g., internal pressure, the height of material’s deposition, and the delivery system. First, the pressure in the caulking gun is generated by hand (manual), while in the cavity pump is more uniform and regulated since it is controlled automatically by the control panel. Second, the height of the material’s deposition is another difference in both processes. In the caulking gun, it is hard to control the height distance between the platform and the nozzle, but this process is controlled automatically through the MATLAB software to the end-effector of the robotic arm. The last point, the delivery system in the caulking gun, directly deposits materials from the tube (there is no delivery hose), while in cavity pump the deposition materials pass through a pump and hose before reaching the nozzle.

## 5. Conclusions

Nowadays, 3DP techniques are increasingly becoming popular for construction applications. This paper presents an investigation on different end-effectors for extrusion printing involved in the 3D printed mortar and concrete geometries utilising a 6DOF industrial robot. Different mix designs, which are used to optimize cementitious mix designs with different delivery systems, were discussed. In addition, how these mixes have been evaluated by numerous trials and tests were explained in detail. Furthermore, it was found that the optimisation of the cementitious mix has a significant impact on the structure created by the robot. The experimental program was executed for the printed specimens in the fresh state and hardened state. The results show that mortar is more suitable and efficient for printing and construction of layers due to the smaller number of voids and less porosity between particles, while the concrete mixes with large particles allow more openings and internal porosity due to trapped air. The lower slump in the mortar is further evidence that the mortar has better performance than the concrete mix for printing applications. Moreover, the sample was printed with fibre to assess their efficiency as a reinforcement in the printed structure. The fibre reinforcement effectively improves the shrinkage, mechanical properties, and ductility of the structure. The investigation into the various layers of the printed specimens has shown that an increase in the number of layers reduces the mechanical strength of the specimens. The printed specimens have been tested in different media of curing, and the results have shown that wet curing improves the mechanical strength of the specimens. Further investigation needs to be done for the curing process, such as using advanced technologies and different media to contribute to AM technology with printing mortar members.

It is recommended that for upcoming studies, the focus should be on predictive models, feedback to reduce the chance of errors, and online sensing to integrate more online coincidences for the robot. Further investigations are necessary into the squeeze flow results using PP fibre for all suggested mixes. It is also recommended that different waiting time intervals between layers could be further investigated, as well as checks of the different heights of the nozzles from the platform.

## Figures and Tables

**Table 1:** Mechanical properties of the polypropylene (PP) fibre that has been used in the mortar mix.

Fibre Type	Length/Diameter	Thickness	Specific Gravity (g/cm^3^)	Tensile Strength (MPa)	Tensile Modulus (GPa)
Polypropylene	6 mm	100 µm	0.91	1300	7.2

**Table 2:** Mortar mix design with a 1:1 ratio (cement to sand) with/without fibre.

Trial No.	Fine Sand (g)	Cement (g)	Coarse Aggregate (g)	Retarder (mL)	Accelerator (mL)	Water (mL)	Superplasticizer (mL)	PP Fibre
T5	375	375	-	2	2.5	125	2.5	0%
T5 *	375	375	-	2	2.5	125	2.5	1%
T8	500	500	-	2.66	3.3	171.5	3.33	0%
T12	375	375	125	2	2.5	125	2.5	0%

* Selected mix using polypropylene fibre.

**Table 3:** Details of the prepared samples.

Sample Description	Number of the Samples	Size of Samples (mm)	Delivery Method
Hollow column	6	(300 × 300)	Cavity pump
Printed without Fibre Reinforced mortar	6	(160 × 40 × 8)	Caulking gun (160 × 35× 10 mm)
	6	(160 × 40 × 16)	
	6	(160 × 40 × 24)	
	6	(160 × 40 × 32)	
Printed with/without Fibre Reinforced mortar	6	(160 × 14 × 12)	Caulking gun (14 mm)
	6	(160 × 14 × 24)	
	6	(160 × 14 × 36)	
	6	(160 × 14 × 48)	
Printed without Fibre Reinforced mortar	6	(160 × 24 × 18)	Caulking gun (20 mm)
	6	(160 × 24 × 36)	
	6	(160 × 24 × 54)	
	6	(160 × 24 × 72)	
Casted prisms	18	(160 × 40 × 40)	Conventional method
Casted cubes	18	(50 × 50 × 50)	Conventional method

**Table 4:** The relative slump value and height of the slump for selected trials.

Trial no.	Height of Slump (mm)	Average Diameter no. 1 (mm)	Average Diameter no. 2 (mm)	Average Relative Slump Value
Trial 5	8.5 ± 0.54	190.63	184.65	2.52
Trial 5 (with 1% PP fibre)	6 ± 0.16	148.27	155.25	1.30
Trial 8	8.8 ± 0.61	199.52	201.84	3.02
Trail 12	12 ± 0.58	201	201	3.04

**Table 5:** The setting time results for the three main trials.

Trial No	Initial Setting Time (min)	Final Setting Time (min)
T5	75 ± 10	120 ± 5
T8	85 ± 14	225 ± 6
T12	75 ± 9	90 ± 4

**Table 6:** Caulking gun extrusion nozzle (Ø14 mm).

Interval Time (min) **	Buildability						Height (mm)					
	1	2	3	4	5	6	1	2	3	4	5	6
3	×	×	×	×	×	×	12	11.5	12	12	12.5	8 *
7	×	×	×	×	×	-	12.5	12	12	12.1	12	-
10	✓	✓	✓	✓	✓	-	12	12.4	12.1	12.1	12.2	-

* Failed extrusion, ** Interval time between layers from mixing until the finish from 1–5 layers.
